# Supplementary material for: Effects of a Brief E-Learning Resource on Sexual Attitudes and Beliefs of Healthcare Professionals Working in Prostate Cancer Care: A Pilot Study
Source: Int J Environ Res Public Health. 2021 Sep 24;18(19):10045. doi: 10.3390/ijerph181910045 (PMC8508566; doi:10.3390/ijerph181910045)
Supplement: Supplementary file 1 [file ijerph-18-10045-s001.zip › ijerph-1355044-supplementary.pdf]

Supplementary Figure S1. Boxplots showing distribution of Sexual Attitude and Beliefs Survey scores for each statement (Q1-Q12) and total survey scores from participants in the pilot study (n=44)

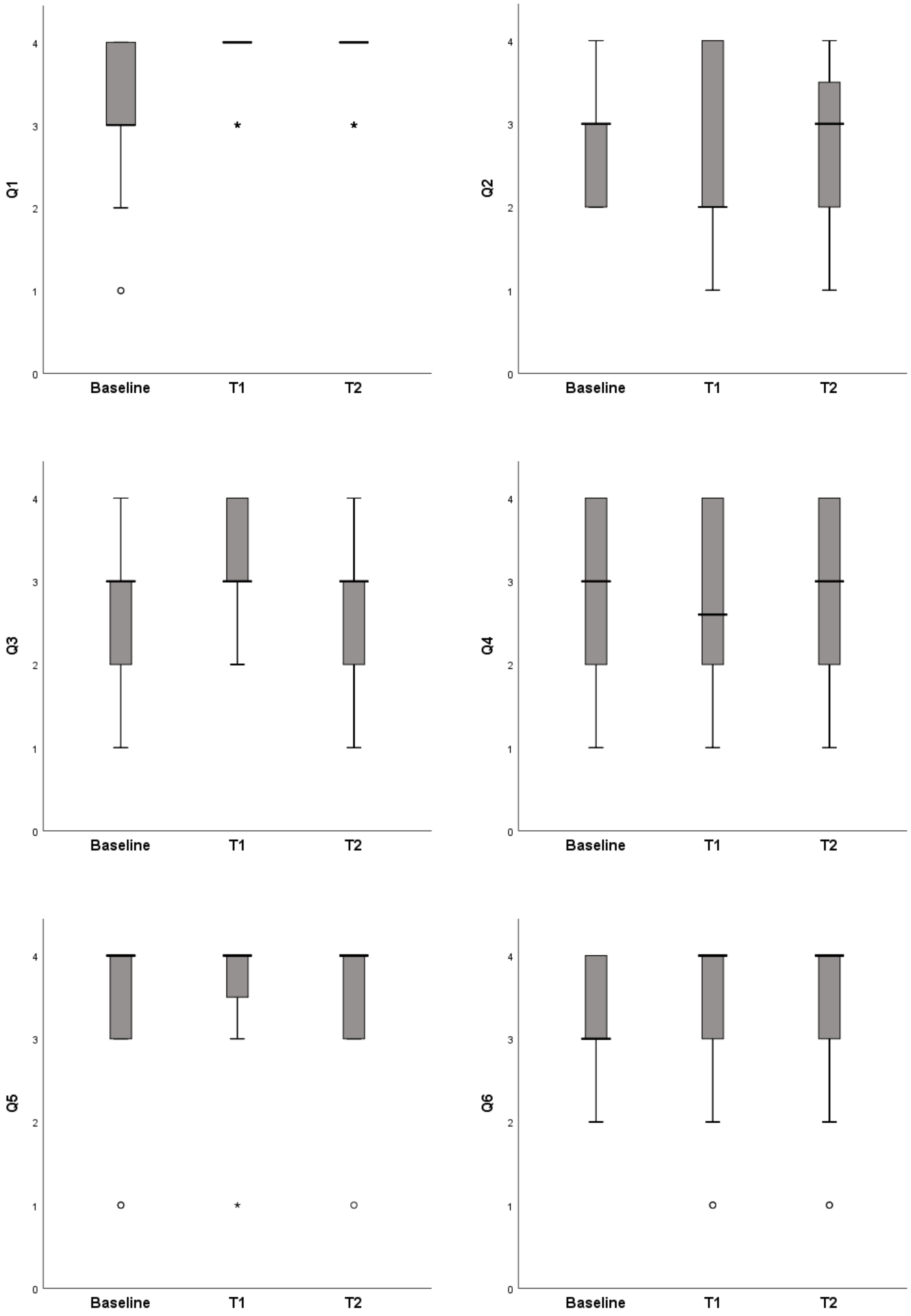

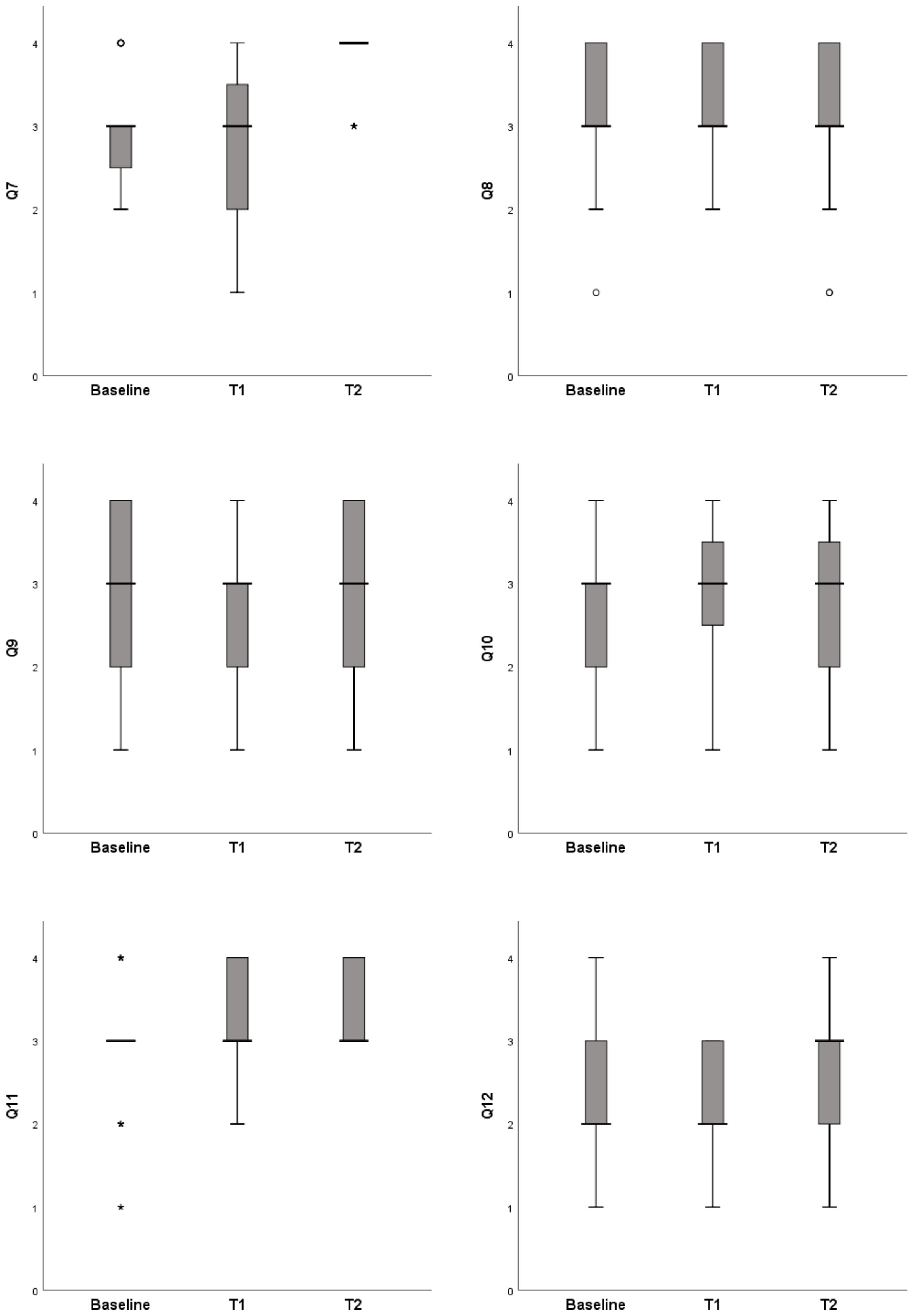
Q7
Q8
Q9
Q10
Q11
Q12
0
1
2
3
4
Baseline
T1
T2

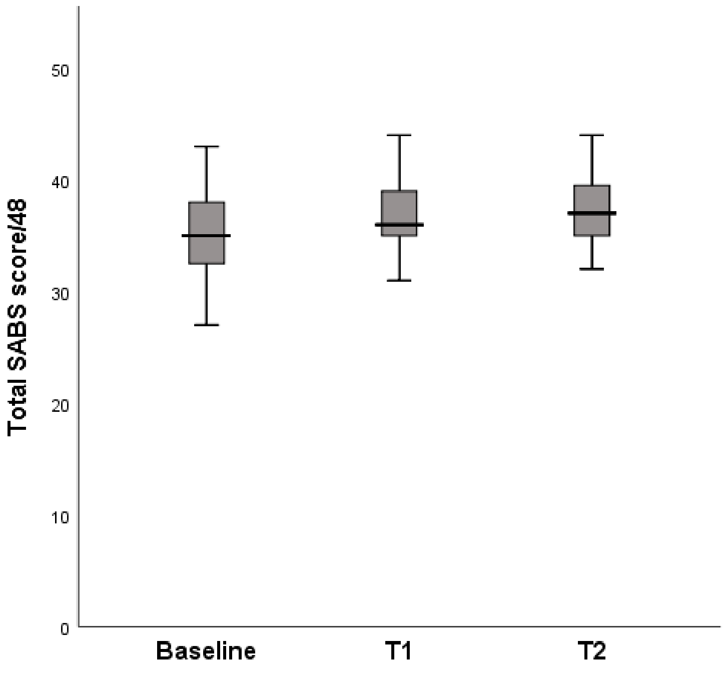

Baseline (immediately prior to completion of the e-learning resource)
T1: Timepoint 1 (immediately post completion of the e-learning resource)
T2: Timepoint 2 (4 weeks after completion of the e-learning resource)

	**Sexual Attitude and Beliefs Survey Items (n=12)**
**1**	I understand how prostate cancer and its treatment might affect men’s sexual wellbeing
**2**	I am uncomfortable talking about sexual issues with men living with prostate cancer*
**3**	I feel confident in my ability to address the sexual concerns of men living with prostate cancer
**4**	Talking about sexual concerns with men living with prostate cancer can ‘open a can of worms’*
**5**	Sexual concerns are an important topic to discuss with men living with prostate cancer
**6**	Discussing sexual concerns with men living with prostate cancer is part of my job
**7**	I make time to discuss sexual concerns with men living with prostate cancer
**8**	Sexual issues should be discussed only if initiated by men living with prostate cancer*
**9**	I find it difficult to talk to older men living with prostate cancer about sexual concerns*
**10**	I know the right language to use when discussing sexual concerns with men living with prostate cancer
**11**	Sexual issues should be discussed with partners of men living with prostate cancer
**12**	Men living with prostate cancer do not expect healthcare professionals to ask about sexual concerns*

Supplementary Table S1. Changes made to the Sexual Attitudes and Beliefs Survey based on testing carried prior to the pilot study (n=66 participants) and internal consistency measures of each item

	Original SABS	Modified SABS (Prostate cancer)	Mean Score (SD)	Corrected item- Total Correltion	Cronbach's Alpha if item deleted
1	I understand how my patients' diseases and treatments might affect their sexuality.	I understand how prostate cancer and its treatment might affect men's sexual wellbeing	3.37 (0.74)	0.63	0.63
2	I am uncomfortable talking about sexual issues.	I am uncomfortable talking about sexual issues with men living with prostate cancer*	2.87 (0.83)	0.34	0.67
3	I feel confident in my ability to address patients' sexual concerns.	I feel confident in my ability to address the sexual concerns of men living with prostate cancer	2.42 (0.82)	0.55	0.65
4	Sexuality is too private an issue to discuss with patients.	Talking about sexual concerns with men living with prostate cancer can 'open a can of worms'*	2.89 (0.93)	-0.11	0.74
5	Discussing sexuality is essential to patients' health outcomes.	Sexual concerns are an important topic to discuss with men living with prostate cancer	3.63 (0.71)	0.48	0.66
6	Giving a patient permission to talk about sexual concerns is a nursing responsibility.	Discussing sexual concerns with men living with prostate cancer is part of my job	3.10 (0.95)	0.62	0.62
7	I make time to discuss sexual concerns with my patients.	I make time to discuss sexual concerns with men living with prostate cancer	2.74 (0.98)	0.63	0.62
8	Sexuality should be discussed only if initiated by the patient.	Sexual issues should be discussed only if initiated by men living with prostate cancer*	3.22 (0.78)	-0.05	0.73
9	When patients ask me a sexually related question, I advise them to discuss the matter with their physician.	I find it difficult to talk to older men living with prostate cancer about sexual concerns* [added item]	2.92 (0.91)	0.24	0.69
10	I am more comfortable talking about sexual issues with my patients than are most of the nurses I work with.	I know the right language to use when discussing sexual concerns with men living with prostate cancer [added item]	2.50 (0.74)	0.55	0.64
11	Hospitalized patients are too sick to be interested in sexuality.	Sexual issues should be discussed with partners of men living with prostate cancer [added item]	3.10 (0.76)	0.28	0.69
12	Sexuality should be discussed only if initiated by the patient.	Men living with prostate cancer do not expect healthcare professionals to ask about sexual concerns*	2.96 (0.74)	0.11	0.70

**Modified SABS (Prostate cancer)**	
Cronbach's Alpha**	**0.69**
Cronbach's Alpha based on standrdised items	**0.69**
N of items	**12**

* indicates a score which is reversed for data analysis. ** Cronbach's Alpha values: 0.91-1.00 = Excellent; 0.81-0.90 = Good; 0.71-0.80 = Good and acceptable; 0.61-0.70 = Acceptable: 0.01-0.60 = Non-acceptable. SD: Standard Deviation
